# Is There a Diagnostic and Prognostic Role for Anti-Nephrin Autoantibodies in Diabetic Nephropathy?

**DOI:** 10.3390/antib14010025

**Published:** 2025-03-12

**Authors:** Han Sean Lee, Henry H. L. Wu, Arvind Ponnusamy, Helen Alderson, Rajkumar Chinnadurai

**Affiliations:** 1 Department of Renal Medicine, Northern Care Alliance NHS Foundation Trust, Salford M6 8HD, UK; han.lee@nca.nhs.uk (H.S.L.); helen.alderson@nca.nhs.uk (H.A.); 2 Renal Research Laboratory, Kolling Institute of Medical Research, Royal North Shore Hospital, The University of Sydney, Sydney, NSW 2065, Australia; hon.wu@sydney.edu.au; 3 Department of Renal Medicine, Lancashire Teaching Hospitals NHS Foundation Trust, Preston PR2 9HT, UK; arvind.ponnusamy@lthtr.nhs.uk; 4 Faculty of Biology, Medicine and Health, The University of Manchester, Manchester M1 7HR, UK

**Keywords:** anti-nephrin, nephrin, diagnostic biomarker, prognostic biomarker, diabetes mellitus, diabetic nephropathy

## Abstract

Diabetic nephropathy (DN) is one of the key causes of end-stage kidney disease worldwide, especially in developed countries. The classic pathogenic development of DN is characterized by microalbuminuria which would progress to nephrotic-range proteinuria and loss of kidney function. The degree of albuminuria is considered an independent risk factor for all-cause mortality in patients with DN. It is now well established that albuminuria stems from disruptions in podocyte structure and function. Podocytes play a major role in the glomerular filtration barrier. The nephrin protein has been identified as a core component of the slit diaphragm in podocytes, and as such, the downregulation of nephrin expression has been described well in various proteinuric glomerulopathies, including DN. Previous studies have shown that the presence of urinary nephrin potentially signifies an early marker of podocyte injury in DN. More recently, there have been increasing bodies of evidence which suggest that circulating autoantibodies targeting nephrin contributes to the pathogenesis of podocytopathies. However, the functional significance of these circulating autoantibodies in patients with DN is not well understood. In this review, we aim to evaluate the significance of nephrin dysregulation in the pathogenesis of DN based on the current available literature and provide an overview on the application of circulating anti-nephrin autoantibodies in relation to its diagnostic as well as prognostic role in podocytopathies, including DN.

## 1. Introduction: The Current State of Diagnostic Approaches in Diabetic Nephropathy

The prevalence of diabetes worldwide is reported to have reached epidemic proportions, and up to 40% of people living with diabetes develop diabetic nephropathy (DN) [1]. As such, DN is expected to continue to be the leading cause of end-stage kidney disease in both developed and developing countries. To reduce the incidence and progression of DN, current guidelines are now focused on developing strategies for allowing early identification and comprehensive management of DN. This includes lifestyle modifications to achieve strict glycemic control as well as managing cardiovascular risk factors, such as optimizing blood pressure control.

Albuminuria progression from microalbuminuria to nephrotic-range proteinuria concurrent with a gradual decline in kidney function are currently recognized as the clinical hallmarks of DN. At present, albuminuria is the only established biomarker for detection of early DN. Furthermore, it is also a key prognostic marker for all-cause mortality, as published studies indicate that the degree of albuminuria positively correlates with progression toward end-stage kidney disease and the development of cardiovascular events [2,3]. When classifying albuminuria, recommendations from Kidney Disease Improving Global Outcomes (KDIGO) are usually adhered to (Table 1). These take into account either an individual’s spot urine albumin–creatinine ratio (ACR) or their 24 h urine albumin excretion rate (AER) [1].

**Figure 1:**
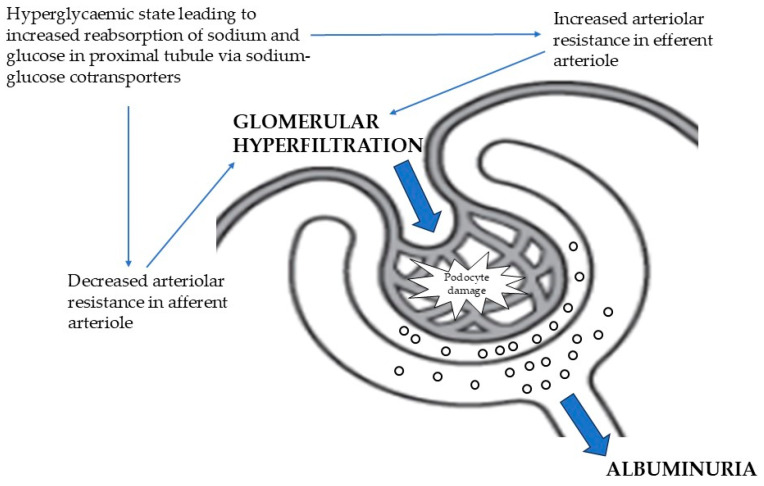
Pathogenesis of albuminuria in diabetic nephropathy.

The most widely accepted pathophysiological process of albuminuria in DN relates to glomerular hyperfiltration driven by increased sodium and glucose reabsorption mediated by sodium-glucose cotransporters during the hyperglycemic state [4]. As reabsorption of sodium and glucose increases in DN, it activates dysregulated tubuloglomerular feedback via the macula densa cells. Subsequently, this increases the vascular resistance in the efferent glomerular arteriole and paradoxically decreases the vascular resistance in the ensuing afferent arteriole, which results in increased glomerular pressure in the state of hyperfiltration. This will, in turn, lead to a cascade of numerous processes of podocyte oxidative stress, detachment, inflammation, and scarring, leading to loss of the glomerular filtration barrier (Figure 1).

The diagnosis of DN in practice has been chiefly based on precisely identifying the clinical manifestations of DN. This would involve taking into account the individual’s albuminuria trajectory alongside their glycemic control historically, as well as the presence of other diabetes-associated macro- or microvascular complications such as diabetic retinopathy. It will then be correlated with an individual’s serum creatinine levels, which is widely used as a marker reflective of the estimated glomerular filtration rate (eGFR). However, evidence from several observational studies identified a sizeable proportion of concurrent non-diabetic nephropathies found among kidney biopsies in patients with diabetes which may require different treatment approaches [5,6,7]. In the largest study to date, Liu et al. retrospectively analyzed the kidney biopsies of 982 type 2 diabetes patients aged above 18 over a 10-year period. It was found that 64% of the patients actually demonstrated non-diabetic kidney diseases [5]. The expanding use of kidney biopsies in diabetic patients with kidney disease remains highly debatable, given the invasiveness of the procedure coupled with the risk of bleeding, which may lead to nephrectomy and death. Hence, there is a growing need for alternative noninvasive diagnostic methods, such as specific serological and urinary markers as well as advanced imaging techniques, which would negate the need for kidney biopsy in most patients.

Numerous alternative diagnostic biomarkers of DN have been studied, and they demonstrated potential as potentially useful noninvasive tools in detecting early kidney dysfunction in DN compared with albuminuria. An example of a promising biomarker in identifying early DN is cystatin C, a protein under the cysteine protease inhibitor family which is freely filtered by the kidney glomeruli due to its low molecular weight. This was initially explored in human subjects by Jeon et al., who recruited a total of 332 type 2 diabetes patients with both normoalbuminuria and varying degrees of albuminuria [8]. Jeon et al. demonstrated that both increasing serum and urinary cystatin C levels significantly correlated with a rising degree of albuminuria. Furthermore, when applying a multivariate logistic regression analysis, both serum and urinary cystatin C were identified as independent factors associated with an eGFR < 60 mL/min/1.73 m^2^ using the conventional creatinine-based Modification of Diet in Renal Disease equation in patients with normoalbuminuria. This contributes forming the theory that the presence of cystatin C in both serum and urine could form the basis of early detection and predict progression of DN even in non-albuminuric patients. However, this study was limited by its relatively small sample size, coupled with its retrospective cross-sectional study design. Thus, the true association between various risk factors relating to patients with DN and the progression of disease remains in question. Larger prospective studies will also be needed to outline the potential application of cystatin C as a future biomarker in clinical practice. Other biomarkers such as serum and urine neutrophil gelatinase-associated lipocalin, plasma kidney injury molecule-1, and plasma tumor necrosis factor alpha were also investigated [9,10,11]. However, the findings to date suggest that these biomarkers only showed a rather modest improvement at best compared with the currently available biomarkers. There were also extremely little to no data to support their use in day-to-day clinical practice.

## 2. Nephrin and the Kidney Filtration Barrier in Diabetic Nephropathy

The discovery of the NPHS1 gene mutation in congenital nephrotic syndrome of the Finnish type over 25 years ago, which encodes the transmembrane protein nephrin, has since revolutionized our understanding of the glomerular filtration barrier at a molecular level [12]. Nephrin, amongst other protein molecules, forms the core component of the structural portion of the slit diaphragm of glomerular podocytes, which mostly determines the size selectivity of the glomerular filtration barrier [13]. Nephrin also forms a complex signaling platform via nephrin tyrosine phosphorylation, which subsequently interacts with other phosphorylated protein tyrosine residues [14,15]. This in turn will influence a podocyte’s cell adhesion and shape, hence regulating the functional and structural integrity of glomerular podocytes. From the studies available, NPHS1 gene mutations were mostly found to be missense mutations, which lead to failure of the transportation of nephrin from the endoplasmic reticulum to the cell surface and a subsequent inability to localize to the slit diaphragm [16].

As DN is an established major cause of podocyte dysfunction and subsequent albuminuria, Aaltonen et al. and Forbes et al. initially sought to explore the role of nephrin in DN pathophysiology using streptozotocin-induced mice kidney models (resulting in diabetes by selectively impairing insulin production in pancreatic beta cells) [17,18]. In both studies, nephrin gene expression in the kidney was identified with immunohistochemistry and quantified using a real-time polymerase chain reaction (PCR). Both studies showed an initial two-to-threefold rise in the expression of nephrin messenger ribonucleic acid (mRNA) in the glomerulus of the diabetic animal models within 6–8 weeks of diabetes onset. Aaltonen et al. also concomitantly replicated these results using a spontaneous non-obese diabetic mice model [17]. Forbes et al. then observed a substantial decline in nephrin expression from 16 weeks of onset which was linked with increasing albuminuria. These findings strongly suggest a connection between nephrin and early DN [18]. It is postulated that the initial increase in expression of nephrin mRNA in early DN represents an initial compensatory surge in nephrin synthesis following podocyte injury. As DN progresses, the sustained and repeated damage to podocytes is reflected by a fall in nephrin expression, as highlighted by Forbes et al. 16 weeks after onset in the animal model.

In addition to the above experimental reports, Doublier et al. was able to translate these findings in the kidney biopsies of 23 patients with both type 1 and type 2 diabetes [19]. Compared with their healthy controls, a meaningful reduction in nephrin expression was demonstrated via immunofluorescence in the human glomeruli of diabetic patients with microalbuminuria. Jim et al. later backed up these findings in a study where the downregulation of nephrin along with other podocyte specific proteins was demonstrated in 15 kidney biopsies of type 2 diabetes patients compared with a control group using immunohistochemistry [20]. Hence, it was hypothesized that nephrin dysregulation may be an important process in the development of albuminuria in DN.

## 3. Urinary Nephrin: A Novel Biomarker for Early Diabetic Nephropathy?

Whilst microalbuminuria remains the gold standard for the early detection and prognostication of DN, its sensitivity and specificity remain questionable with the current body of evidence. Microalbuminuria is also linked to other pathological processes such as urinary tract infections, cardiovascular diseases, and any acute illness leading to hemodynamic stress. Therefore, there is still a need to establish diagnostic and predictive markers of early DN manifestations outside of albuminuria. It is widely accepted that podocyte injury in DN leads to excretion of its various protein fragments into urine. As such, various urinary biomarkers have been a focus of research to promote the early identification, prevention, and treatment of DN.

Aaltonen et al. first reported the presence of the nephrin protein in urine through immunoblotting in an initial experimental study using streptozotocin-induced diabetic rats in Finland [17]. Interestingly, nephrin was identified earlier than albumin in urine during the course of diabetic kidney disease. Subsequently, in an early human study by Patari et al., the presence of urinary nephrin using the western Blotting method was examined in type 1 diabetes patients with both non-albuminuria and albuminuria against healthy controls. It was found that 30% of the type 1 diabetes patients displayed the presence of nephrin in their urine even without the presence of albuminuria, whereas nephrinuria’s presence was not demonstrated in the non-diabetic healthy subjects [21]. This supported their hypothesis that urinary nephrin could be utilized as an early marker of DN compared with microalbuminuria.

Jim et al. also examined the association of urinary nephrin with DN in a small cohort of type 2 diabetes patients with varying degrees of albuminuria [20]. Urinary nephrin was measured using enzyme-linked immunosorbent assays (ELISAs). It was demonstrated that nephrinuria was found in all patients with microalbuminuria and 54% of diabetic patients without microalbuminuria. Urinary nephrin levels also showed a strong positive correlation with albuminuria and were positively correlated with a reduction in eGFR. These results confirm that nephrinuria can be seen in early DN before microalbuminuria and urinary nephrin level increases in overt disease.

More recently, the significance of urinary nephrin as an early biomarker of DN is further corroborated in a cross-sectional study by Kostovska et al. and a case–control study by Veluri et al. which included type 2 diabetes patients with or without a diagnosis of DN and matched healthy controls [22,23]. Both studies showed that urinary nephrin as a biomarker of early DN has a high diagnostic sensitivity and specificity of up to 100% and 88%, respectively. Nephrinuria also has a predictive probability of 96% in subjects with DN. This study also verified the significant negative correlation of urinary nephrin levels with eGFR, indicating this as an early predictor of poor kidney function.

Whilst all human studies to date indicated potential for urinary nephrin as a favorable early biomarker for pre-clinical DN in comparison with microalbuminuria, all reported studies were limited by their small sample sizes. The investigators also acknowledged that the study design for all of these reported studies could neither demonstrate a causal mechanism of nephrinuria nor consistently predict the future progression of DN in patients with pre-clinical DN. Moving forward, a large prospective study involving patients with non-albuminuric diabetes may address these knowledge gaps. For such validation studies, ideally, a follow-up period of at least 10 years from the onset of diabetes would be required.

## 4. Anti-Nephrin Autoantibodies and Their Role in Diabetic Nephropathy

In their landmark study published in 2021 in the Journal of the American Society of Nephrology, Watts et al. first detected circulating anti-nephrin autoantibodies in a subset of children and adults with biopsy-proven minimal change disease (MCD), a well-known cause of nephrotic syndrome [24]. MCD is characterized by the absence of a noticeable pathological change in kidney glomeruli when observed under light microscopy. However, ultrastructural analyses using electron microscopy will reveal extensive damage to podocytes in the form of podocyte foot effacement and fusion [25]. Previous observational studies published as early as 1954 demonstrated the capability of plasma taken from subjects with nephrotic syndrome to induce proteinuria in non-nephrotic subjects [26]. Therefore, there was considerable focus over the years to identify the presence of specific circulating factors derived from lymphocytes in the pathogenesis of MCD.

The pivotal discovery by Watts et al. was formed on the basis of previous study findings in animal models where circulating antibodies targeting nephrin in the podocyte slit diaphragm induced massive proteinuria [27,28]. More importantly, these circulating antibodies were either markedly reduced or absent during partial or complete remission of nephrotic syndrome. In the same animal models, these anti-nephrin antibodies were found to result in a redistribution of nephrin away from the podocyte slit diaphragm, which leads to podocyte dysfunction and subsequent proteinuria. These findings have not only provided us with initial understanding into the causative role of anti-nephrin autoantibodies in the development of podocytopathies but also justified the efficacy of B cell depletion therapies, which are being studied in clinical trials involving patients with nephrotic syndrome [29,30].

In a recent large, multicenter study by Hengel et al., the presence of anti-nephrin autoantibodies was validated in a significant proportion of adults and children with podocytopathies such as MCD, primary focal segmental glomerulosclerosis (FSGS), and idiopathic nephrotic syndrome (INS) [31]. The prevalence of these autoantibodies, which were detected using a sensitive hybrid immunoprecipitation ELISA, was reported to be as high as 69% in adults with untreated active MCD and 90% in children with untreated active INS. In addition, the levels of anti-nephrin autoantibodies also firmly correlated with proteinuria and disease activity in anti-nephrin autoantibody-associated podocytopathies. This was evidenced using longitudinal measurements of anti-nephrin autoantibodies in select subcohorts of 18 children with INS, 13 adults with MCD, and 5 adults with primary FSGS, founding that a negative anti-nephrin autoantibody titre was predictive of remission (defined by a urine protein-to-creatinine ratio of <0.3 g/g) and a reduction in proteinuria to a urine protein-to-creatinine ratio of <3.5 g/g in over 90% of subjects. This study then substantiated a causal relationship between anti-nephrin autoantibodies and podocyte dysfunction using an experimental mice model actively immunized with recombinant murine nephrin. Following immunization, these mice developed circulating anti-nephrin autoantibodies and, subsequently, nephrotic syndrome, mimicking the clinicopathological features of MCD. Through electron microscopy, it was revealed that gold labeling of mouse immunoglobulin (IgG) was isolated at the slit diaphragms, especially in areas of diffusely effaced podocyte foot processes. Furthermore, proteomic and phosphoproteomic analyses of glomeruli in immunized mice showed increased nephrin tyrosine phosphorylation, which altered the downstream signaling pathways, leading to actin assembly, cytoskeletal reorganization, and nephrin endocytosis. These changes subsequently led to structural alterations in keeping with diffuse effacement of podocyte foot processes.

Overall, the seminal paper by Hengel et al. provided a robust case in which circulating anti-nephrin autoantibodies are not merely markers of disease activity but also actively contribute to the pathogenesis of podocytopathies. This insight strengthened our focus toward targeting specific kidney-specific autoantibodies rather than the histological description in diagnosing glomerular diseases, which also suggests the potential for novel, targeted therapies [32]. This is supported by previous breakthrough findings, such as autoantibodies against the phospholipase A2 receptor (PLA2R) in primary membranous nephropathy and autoantibodies against the non-collagenous domain of the type IV collagen on the glomerular basement membrane (GBM) in anti-GBM disease [33,34].

Subsequently, Shirai et al. also presented evidence of the predictive role of preexisting circulating anti-nephrin autoantibodies in a small subset of Japanese post-transplant patients with recurrent FSGS [35]. Similar to the animal model in the study by Hengel et al., the kidney transplant biopsies in all 11 patients following the recurrence of FSGS showed deposition of IgG co-localized with nephrin and increased nephrin phosphorylation. Additionally, a significant positive correlation with their respective serum anti-nephrin antibody levels was also confirmed.

Despite prior compelling evidence of nephrin dysregulation in DN and the active role of circulating anti-nephrin autoantibodies leading to podocyte disruption, there is a glaring paucity of data currently relating to the applications of anti-nephrin autoantibodies in DN. To date, only one study, published by Aaltonen et al. in 2007, investigated the prevalence and role of autoantibodies with nephrin in patients with type 1 diabetes [36]. In this study, the stored serum samples of 66 children and adolescents diagnosed with type 1 diabetes along with 96 non-diabetic control subjects were measured for anti-nephrin autoantibodies over a 10-year period since the initial diagnosis of diabetes using a radioimmunoprecipitation assay. Aaltonen et al. identified 24% of diabetic patients testing positive for anti-nephrin autoantibodies at the time of diagnosis and 44% providing at least one positive sample over the 10-year study’s follow-up period. When assessing the subset of diabetic patients with kidney pathology and microalbuminuria, only 4 out of the 14 patients tested positive for anti-nephrin autoantibodies at any point. Whilst it was noted that the mean duration of manifestation of microalbuminuria was shorter in patients with a positive anti-nephrin autoantibody titre at any point compared with a negative test, this difference was not statistically significant. Therefore, the investigators could not determine any significant causative correlation between circulating anti-nephrin autoantibodies and DN pathophysiological progression here. It was postulated at the time that this negative finding was explained by the screening method employed in this study; the autoantibodies against epitopes of only the intracellular part of nephrin were measured, in which they may not have played a role in the pathogenesis of DN.

## 5. Conclusions

**Figure 2:**
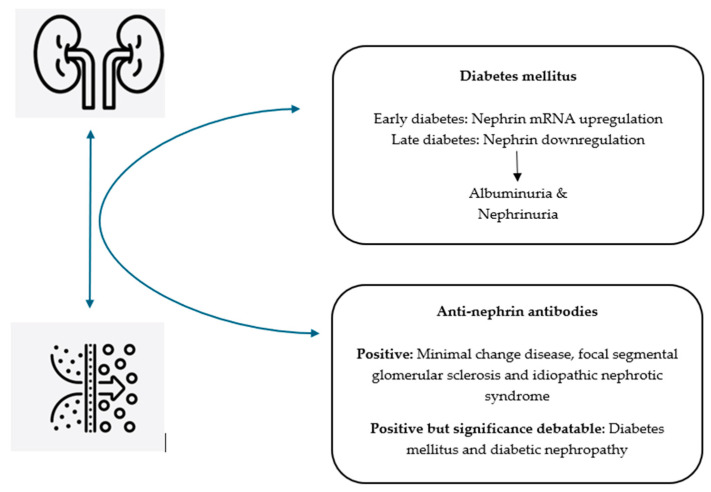
Currently postulated roles of nephrin and anti-nephrin autoantibodies in the pathophysiology of diabetes mellitus and proteinuric glomerulopathies including diabetic nephropathy (mRNA = messenger ribonucleic acid) [10,11,12,13,15,16,22,27].

Identifying nephrin as an integral unit in the glomerular filtration barrier has transformed our understanding of the pathophysiological process of podocytopathies. This new knowledge would bring novel value for influencing the future applications of nephrin-associated markers in clinical practice. Whilst DN continues to be the principal cause of podocytopathy, there remains a major knowledge gap in the role of nephrin and anti-nephrin autoantibodies as an early diagnostic and prognostic marker to assess kidney disease progression. The potential of utilizing this marker to reliably differentiate DN from other proteinuric glomerulopathies such as minimal change disease, FSGS, and INS remains to be elucidated. Nevertheless, the published preliminary small-scale studies provided evidence of nephrin dysfunction in DN, resulting in the subsequent breakdown and release of nephrin via urine (Figure 2). Coupled with the milestone discovery relating to the use of circulating anti-nephrin autoantibodies in proteinuric glomerulopathies (Figure 2), these findings can form the foundation for future research into an alternative causative mechanism of podocytopathy in DN, which could potentially revolutionize our insights into its treatment approach.

## Figures and Tables

**Table 1:** KDIGO classification of albuminuria in chronic kidney disease.

Albuminuria Category	Albumin Excretion Rate (AER)	Albumin–Creatinine Ratio (ACR)	
		mg/mmol	mg/g
Normal to mildly increased (A1)	<30	<3	<30
Moderately increased (A2)	30–300	3–30	30–300
Severely increased (A3)	>300	>30	>300

## Data Availability

Not applicable.

## Abbreviations

The following abbreviations are used in this manuscript:

DN	Diabetic nephropathy
eGFR	Estimated glomerular filtration rate
ELISA	Enzyme-linked immunosorbent assay
FSGS	Focal segmental glomerulosclerosis
GBM	Glomerular basement membrane
IgG	Immunoglobulin G
INS	Idiopathic nephrotic syndrome
MCD	Minimal change disease
mRNA	Messenger ribonucleic acid
PCR	Polymerase chain reaction
PLA2R	Phospholipase A2 receptor
